# Does Vitamin D play a role in the management of Covid-19 in Brazil?

**DOI:** 10.11606/s1518-8787.2020054002545

**Published:** 2020-05-22

**Authors:** Helena Ribeiro, Keila Valente de Souza de Santana, Sofia Lizarralde Oliver, Patricia Helen de Carvalho Rondó, Marcela Moraes Mendes, Karen Charlton, Susan Lanham-New

**Affiliations:** I Universidade de São Paulo Faculdade de Saúde Pública Departamento de Saúde Ambiental São PauloSP Brasil Universidade de São Paulo. Faculdade de Saúde Pública. Departamento de Saúde Ambiental. São Paulo, SP, Brasil; II Universidade de São Paulo Faculdade de Saúde Pública Programa de Pós-Graduação em Saúde Global e Sustentabilidade São PauloSP Brasil Universidade de São Paulo. Faculdade de Saúde Pública. Programa de Pós-Graduação em Saúde Global e Sustentabilidade. São Paulo, SP, Brasil; III Universidade de São Paulo Faculdade de Saúde Pública Departamento de Nutrição São PauloSP Brasil Universidade de São Paulo. Faculdade de Saúde Pública. Departamento de Nutrição. São Paulo, SP, Brasil; IV University of Surrey School of Biosciences and Medicine Faculty of Health and Medical Sciences GuildfordSurrey United Kingdom University of Surrey. School of Biosciences and Medicine. Faculty of Health and Medical Sciences. Department of Nutritional Sciences. Guildford, Surrey, United Kingdom; V University of Wollongong School of Medicine Faculty of Science Wollongong Australia University of Wollongong. School of Medicine. Faculty of Science. Medicine and Health. Nutrition and Dietetics. Wollongong, Australia

**Keywords:** Coronavirus Infections, prevention & control, Vitamin D, Review

## Abstract

The study discusses the possible role of adequate vitamin D status in plasma or serum for preventing acute respiratory infections during the Covid-19 pandemic. Our arguments respond to an article, published in Italy, that describes the high prevalence of hypovitaminosis D in older Italian women and raises the possible preventive and therapeutic role of optimal vitamin D levels. Based on literature review, we highlight the findings regarding the protective role of vitamin D for infectious diseases of the respiratory system. However, randomized controlled trials are currently lacking. Adequate vitamin D status is obtained from sun exposure and foods rich in vitamin D. Studies in Brazil have shown that hypovitaminosis D is quite common in spite of high insolation. Authors recommend ecological, epidemiological and randomized controlled trials studies to verify this hypothesis.

## INTRODUCTION

On March 25, 2020, doctors Isaia and Medico^a^, from Università degli Studi of Turin, Italy, released study, still in pre-print form, based on literature review, titled *Possible preventive and therapeutic role of vitamin D in the management of the Covid-19 pandemic.* In the manuscript^1^, the authors relate hypovitaminosis D to the prevention and treatment of Covid-19, in association with other essential preventive measures. According to the authors:

Sulla base di numerose evidenze scientifiche e di considerazioni epidemiologiche, sembra che il raggiungimento di adeguati livelli plasmatici di Vitamina D sia necessario anzitutto per prevenire le numerose patologie croniche che possono ridurre l’aspettativa di vita nelle persone anziane, ma anche per determinare una maggiore resistenza all’infezione COVID-19 che, sebbene con minore evidenza scientifica, può essere considerata verosimile. (Isaia and Medico, 2020, p.1)^a^

Reference studies show the role of optimal vitamin D levels in the prevention and control of severe respiratory infections.

Since the authors collaborate in an international research aimed to investigate vitamin D status in older women from four continents^b^, contributions to this topic are being made within the context of the Covid-19 pandemic. The rationale is based on evidence of the potential protective role of vitamin D in several diseases.

### VITAMIN D AND THE HEALTH OF THE OLDER POPULATION

Adequate vitamin D status can be obtained from adequate sun exposure and foods rich in vitamin D. Recently, vitamin D deficiency has been considered a global and widespread health concern at all ages^2^. Older people are the most prone to hypovitaminosis D due to (a) low sun exposure when homebound or institutionalized, (b) reduced skin capacity to convert dehydrocholesterol into cholecalciferol, (c) changes in appetite and diet predisposing suboptimal vitamin D intake accompanied by reduced gastrointestinal absorption, (d) polypharmacy including medications that interfere in vitamin absorption or metabolism and (e) impairment of renal function^3^.

Changes or deficiencies in the activation and control of vitamin D absorption result in organic disorders that may evolve to important pathologies such as rickets in children and osteomalacia or osteoporosis in adults^3^. A body of analyses from epidemiological evidence shows an association between vitamin D deficiency and increased risk of diabetes, cardiovascular diseases, osteoporosis, osteoarthritis, Alzheimer’s/dementia, myopia, macular degeneration, multiple sclerosis and some types of cancer^7,8^. However, the impact of vitamin D supplementation on these outcomes in randomized controlled trials (RCT) has not been reported.

### VITAMIN D AND COVID-19

Researchers have been studying the protective potential of vitamin D against diseases and health-related problems for many years, but here we highlight the protective role of vitamin D for infectious diseases of the respiratory system. In their review, Isaia and Medico^1^ cite the study of Bischoff-Ferrari^9^, in which reduced concentrations of 25 (OH)D increase the risk of osteoporosis in older adults, being also associated with tumors, cardiovascular diseases, chronic autoimmune respiratory diseases, diabetes mellitus, neurological diseases and hypertension. As highlighted by Isaia and Medico^1^, “these pathologies potentially cause higher mortality, especially if these patients are affected by Covid-19” (p.2*),* but this has not yet been proven.

Experimental studies using cell lines and mice have been conducted to elucidate the pulmonary activation of vitamin D3 and its preventive effect against interstitial pneumonia^10^. Findings suggest that vitamin D is activated in the lungs and that its dietary intake can prevent interstitial pneumonia by suppressing pulmonary fibrosis. Another study^11^ found that vitamin D injected into veins of rats, in pharmacological doses, relieved acute lung injury induced by lipopolysaccharide (LPS). Sabetta et al.^12^ hypothesize that supplementation with vitamin D, to increase concentrations in the general population, above 38 ng/ml, would result in significant health benefit, by reducing the burden of viral infections of the respiratory tract in healthy adults living in temperate climates. However, this is based solely on speculation over the results of animal testing.

Isaia and Medico^1^, based on these and other studies, address hypovitaminosis D and the epidemiology of Covid-19 that has been affecting Italy. They argue that Italy is a country with a high proportion of older people, and that 76% of older Italian women show vitamin D deficiency, without significant regional differences^13^. Nevertheless, Dr. Isaia informed: “*I have to underline that it is not a scientific paper, but only a literature review and an invitation to reach normal levels of vitamin D in ageing population, and in particular in Coronavirus patients*” (Isaia, March 28, 2020, personal communication to these authors).

Recently, *Sociedade Brasileira de Endocrinologia e Metabologia (SBEM – Brazilian Society of Endocrinology and Metabolism) and Associação Brasileira de Avaliação Óssea e Osteometabolismo (ABRASSO – Brazilian Association of Bone Assessment and Osteometabolism)* published a note^c^ highlighting that no indication for vitamin D supplementation was approved for effects other than bone health. In this note, they mention an article of the Italian newspaper La Reppublica, regarding a study conducted at Turin University, which suggested that vitamin D could act in the prevention and treatment of Covid-19. Without mentioning the author’s name, the note informs that this study has not been published in a scientific journal and that relevant data were not cited, including the number and age of participants and their 25 (OH) D levels.

We believe this debate is based mainly on news of a non-scientific periodical and gained importance in social media, erroneously motivating self-administration of high doses of vitamin D. Meanwhile, the Brazilian president announced that vitamin D would be exempt of import tax as a measure to combat coronavirus.

### OTHER STUDIES THAT POTENTIALLY SUPPORT THE ROLE OF VITAMIN D

Ergocalciferol (D_2_) and cholecalciferol (D_3_) are among the most important compounds that make up vitamin D. Ergocalciferol or vitamin D_2_ results from ultraviolet irradiation of sunlight on ergosterol^5^. Pre-vitamin D_3_ or cholecalciferol originates from a photochemical cleavage suffered by the cutaneous precursor of vitamin D, 7-dehydrocholesterol, when exposed to ultraviolet radiation^5,14^. Currently, there is no universal consensus on the optimal concentrations for plasma/serum 25-hydroxyvitamin D [25 (OH) D]. In the USA, vitamin D deficiency is defined as a plasma/serum 25 (OH) D status below 12 ng/ml (30 nmol/L) and insufficiency of 25 (OH) D status below 20 ng/ml (50nmol/l)^15^. In the UK, vitamin D deficiency is defined as a plasma/serum 25 (OH) D status below 10 ng/ml (25 nmol/L)^16^. Cannel et al.^17^ suggest 50 ng/ml is protective against viral respiratory infections, particularly in obese, older population, and those with dark skin. This finding, however, is not based on the results of a vitamin D RCT in humans. Dancer et al.^18^ referred that patients with, and at risk of, acute respiratory distress syndrome, are highly likely to be deficient in vitamin D, based on evidence from human, murine and vitro experimental studies.

Ultraviolet B (UVB) radiation is the main source of the vitamin, providing around 80%. Foods such as salmon and other oily fish, cod liver oil, egg yolk, milk and sun-dried mushrooms are natural sources of vitamin D. Vitamin D supplementation and fortified foods such as butter and milk are widely used in countries at high latitude with long winters such as the USA. This is not the case in Brazil. The time of year, latitude, skin color, age and use of sunscreen influence skin production^19,4^. The widespread prevalence of hypovitaminosis D can also be attributed to environmental factors, such as air pollution, which reduces sunlight exposure^2,20^.

Exposure to chemical products and microparticles, dispersed in the urban atmosphere, and tobacco smoke can disturb biochemical pathways and cause harmful consequences, such as vitamin D deficiency^21^. These factors are directly or indirectly implicated in the interruption of the endocrine system of vitamin D and in the decrease in serum levels of two main metabolites: Calcifediol and Calcitriol^21^. While the shade of trees allows sufficient transmission of UVB for vitamin synthesis, that of buildings and glass windows attenuates biologically significant wavelengths^22^. The time spent in outdoor activities by the population is directly and indirectly related to the existence of green areas, levels of air pollution, safety, and urban mobility.

Behavioral and cultural factors that influence the way of life can also reduce the ability to synthetize vitamin D by sunlight exposure. People with long working hours indoors and ethnic minority groups with formal requirement for heavy and covered clothing style have reduced capacity to synthesize vitamin D from UVB radiation^23,24^. Darker skinned ethnic groups are the most affected by the lack of UVB availability for the production of vitamin D by the skin^24^.

With the exception of countries located at high latitudes, most individuals would obtain sufficient levels of vitamin D through sunlight exposure. However, the prevalence of vitamin D deficiency has also been reported in low latitude regions. In Brazil, a study conducted in the city of São Paulo found that 72% of institutionalized older adults and 43.8% of older people attending outpatient clinics showed lower levels of vitamin D^3^ than recommended, contradicting the inference that in Brazil the population is vitamin D-replete due to the high degree of insolation^3^.

Results of our study on vitamin D levels, in Araraquara, SP, called *Morada do So*l (House of the Sun), with 101 community-dwelling healthy women aged 35 years or older, show that 15.8% and 1% had vitamin D insufficiency and deficiency, respectively. However, 25% of Brown women had insufficiency, according to IOM guidelines^15^, which is relevant to many Brazilians. Higher prevalence of vitamin D insufficiency was also found in women who worked in health and aesthetics services. This shows that even in tropical climates, a person’s occupation may influence sun exposure and vitamin D concentrations. In our study, vitamin D levels were also inversely correlated with body mass index and waist circumference. Obesity is linked to vitamin D insufficiency because it hinders the bioavailability of vitamin D^25^ and can lead to health complications, such as the development of some types of diabetes.

Many factors worsen vulnerability of populations to viral diseases, such as comorbidities that might be associated with vitamin D deficiency. Comorbidities have also been associated with higher risk of hospitalization and deaths among those infected by Covid-19, increasing the demand for beds and intensive care units, at a time of shortage.

## FINAL CONSIDERATIONS

Hypovitaminosis D constitutes an additional risk to respiratory tract infections and to the response of the immune system among the Brazilian population, despite high insolation, even in winter. Further studies are urgently needed to investigate whether vitamin D status or supplementation might decrease severity of Covid-19, particularly vitamin D randomized controlled trials. Regular sunlight exposure is a preventive measure against vitamin D deficiency and can prevent diseases related to it. The urban population, especially the older one, tends to be less exposed to sunlight, due to many factors^24^. Thus, people must achieve and maintain adequate serum 25 (0H) D levels in a period of social distancing and self-isolation. This might be obtained through lifestyle strategies, and a healthy balanced diet. Considering the pandemic risk, the WHO and health authorities’ recommendations for staying at home must be followed. For those who do not live in houses with gardens, sunlight exposure can be obtained from open windows or balconies. Only if that is not possible, should an oral vitamin D supplement be considered at SACN/IOM recommended doses to maintain adequate vitamin D levels for general good health. We strongly advise against high vitamin D supplements (e.g. 10,000IU per day) being currently recommended^26^.

Adequate vitamin D status may play a role in prevention and management of respiratory tract infections, which might include the Covid-19 pandemic, especially among the older population and health professionals worldwide. We highly recommend ecological, epidemiological and RCT studies to verify this hypothesis.

## Footnotes

a Based on numerous scientific evidences and epidemiological considerations, it seems that the achievement of adequate plasma levels of Vitamin D is necessary first of all to prevent the numerous chronic pathologies that can reduce life expectancy in elderly, but also to determine a greater resistance to covidCovid-19 infection which, although with less scientific evidence, can be considered similar. (our translation)

b University Global Partnership Network. Healthy living for healthy ageing. Surrey, UK UGPN; 2017 Award. Available from: https://ugpn.org

c Sociedade Brasileira de Endocrinologia e Metabologia. Nota de esclarecimento: Vitamina D e COVID-19. Rio de Janeiro: SBEM; 2020 [cited 2020 Apr 1]. Available from: https://www.endocrino.org.br/nota-de-esclarecimento-vitamina-d-e-covid-19/
